# Ethnobotanical study of some of mosquito repellent plants in north-eastern Tanzania

**DOI:** 10.1186/1475-2875-7-152

**Published:** 2008-08-07

**Authors:** Eliningaya J Kweka, Franklin Mosha, Asanterabi Lowassa, Aneth M Mahande, Jovin Kitau, Johnson Matowo, Michael J Mahande, Charles P Massenga, Filemoni Tenu, Emmanuel Feston, Ester E Lyatuu, Michael A Mboya, Rajabu Mndeme, Grace Chuwa, Emmanuel A Temu

**Affiliations:** 1 Tropical Pesticides Research Institute, Division of Livestock and Human Disease Vectors Control, P.O. Box 3024, Arusha,Tanzania; 2 KCM College of Tumaini University, P.O. Box 2240, Moshi, Tanzania; 3 Tanzania Wildlife Research Institute, P.O. Box 661, Arusha, Tanzania; 4 Joint Malaria Programme, P.O. Box 2228, Moshi, Tanzania; 5 Institute of Tropical Medicine, Nagasaki University, 1-12-4 Sakamoto, Nagasaki, Japan

## Abstract

**Background:**

The use of plant repellents against nuisance biting insects is common and its potential for malaria vector control requires evaluation in areas with different level of malaria endemicity. The essential oils of *Ocimum suave *and *Ocimum kilimandscharicum *were evaluated against malaria vectors in north-eastern Tanzania.

**Methodology:**

An ethnobotanical study was conducted at Moshi in Kilimanjaro region north-eastern Tanzania, through interviews, to investigate the range of species of plants used as insect repellents. Also, bioassays were used to evaluate the protective potential of selected plants extracts against mosquitoes.

**Results:**

The plant species mostly used as repellent at night are: fresh or smoke of the leaves of *O. suave *and *O. kilimandscharicum *(Lamiaceae), *Azadirachta indica *(Meliaceae), *Eucalyptus globules *(Myrtaceae) and *Lantana camara *(Verbenaceae). The most popular repellents were *O. kilimandscharicum *(OK) and *O. suave *(OS) used by 67% out of 120 households interviewed. Bioassay of essential oils of the two Ocimum plants was compared with citronella and DEET to study the repellence and feeding inhibition of untreated and treated arms of volunteers. Using filter papers impregnated with Ocimum extracts, knockdown effects and mortality was investigated on malaria mosquito *Anopheles arabiensis *and *Anopheles gambiae*, including a nuisance mosquito, *Culex quinquefasciatus*. High biting protection (83% to 91%) and feeding inhibition (71.2% to 92.5%) was observed against three species of mosquitoes. Likewise the extracts of Ocimum plants induced KD_90 _of longer time in mosquitoes than citronella, a standard botanical repellent. Mortality induced by standard dosage of 30 mg/m^2 ^on filter papers, scored after 24 hours was 47.3% for OK and 57% for OS, compared with 67.7% for citronella.

**Conclusion:**

The use of whole plants and their products as insect repellents is common among village communities of north-eastern Tanzania and the results indicate that the use of *O. suave *and *O. kilimandscharicum *as a repellent would be beneficial in reducing vector biting. The widespread use of this approach has a potential to complement other control measures.

## Background

Recently, the environmental friendly and biodegradable natural insecticides of plants origin have been receiving attention as an alternative green measure of control of arthropods of public health importance [1]. More than two billion people, mostly in tropical countries, are at risk from mosquito-borne diseases, such as malaria, dengue, haemorrhagic fever and filariasis [2]. Malaria accounts for 310 – 515 million clinical episodes with 1.5 – 3.0 million deaths per year, 90% of which occur in sub-Saharan Africa [2]. The burden of malaria has been increasing due to development of resistance against both anti-malaria drugs and insecticides, complex social structures, and rapid environmental changes that have intensified in the last decade [1,3]. Consequently, there is no single method of malaria control that is completely effective in high transmission areas [4-6]. Even the most widely tested interventions, using bed nets treated with pyrethroid insecticides, have proven difficult to implement correctly because of problems related to equity, accessibility, user compliance and insecticide resistance [7,8]. For example, in western Kenya, the most important reasons for non-adherence to use of ITNs was the disruption of sleeping patterns due to visitors, funerals, house constructions and other events [9]. Other concerns included fear of the insecticide, which is thought by some, to be a toxic drug used for family planning purposes [10]. Insect repellents play an important role in reducing man-vector contact [11]. Repellents of plant origin have been used for medicinal purposes for a long time because they do not pose hazards of toxicity to human or domestic animals and are easily biodegradable [11,12]. Compared to other synthetic compounds, natural products are presumed to be safer for human use [13], justifying therefore a broad search for eco-friendly biological materials to be used for the control of vectors of medical importance.

The chemical contents extracted from plant materials can be useful as repellents, larvicides, oviposition attractants, insect growth hormone regulators and deterrent agents [11,14,15]. Plant products have been used in many parts of the world for killing or repelling mosquitoes either as extracts or as whole plant [16]. Certain natural products have been investigated for repellent activity against mosquitoes [17,18]. *Ocimum kilimandscharicum *(OK) and *Ocimum suave *(OS) have been reported to possess repellent properties against mosquitoes [16]. The repellent action of plant parts or oil extracts from Ocimum species have been evaluated against Afro-tropical mosquitoes [16,19]. Quelling, an insect repellent produced in Asia, derived from extracts of the eucalyptus and lemon grass has been evaluated against mosquitoes [20]. Essential oil obtained from *Vitex negudo *and flowers from *Lantana camara *have shown repellence activities against *Aedes aegypti *[20,21].

This study present an account of plants used as insect repellents in north-eastern Tanzania and evaluates the feeding inhibition, knockdown effect and mortality effect of two common repellents, *Ocimum suave *and *Ocimum kilimandscharicum *plants against *An*. *gambiae *ss, *An. arabiensis *and *Cx*.*quinquefasciatus*.

## Materials and methods

### Ethnobotanical survey area

The surveys were taken from January to March 2006, at Lower Moshi villages (37°20' E, 3°21' S; 750 M above sea level), located 19 km South of Moshi town, on the foot slopes of Mount Kilimanjaro. The area is characterized by a wet tropical climate with main rain season from March to May, short rain season from November to December and dry season from August to October. The selected villages were Mabogini, Rau Kati, Chekereni and Mtakuja with estimated population of 20,614 [22]. Main ethnic groups inhabiting the study area include the Masai, Pare and Chagga tribes. The objectives and importance of the study, together with methods of data collection were introduced to the community through a village meeting. Standard questionnaires were dispensed by trained staffs to the randomly selected households for one week in each village. Before the interview, the head of household was asked to read a letter of consent explaining the purpose of study and participation was voluntary. The questioner used was designed to capture information on species of plants used as mosquito repellents, method of application, frequency of use, source of knowledge on plants and other means of protection used.

### Mosquitoes

All laboratory tests were conducted using (F1, F2 or F3 generations) female mosquitoes; colonies of *An. gambiae *s.s. (from Kisumu, Kenya), *An. arabiensis *and *Cx. quinquefasciatus *were maintained in the laboratory. *Anopheles gambiae *is highly anthropophilic, whereas *An. arabiensis *is highly zoophilic and partly anthropophilic. *Culex quinquefasciatus *is a nuisance mosquito exhibit catholic feeding behaviour.

### Extraction of volatile oils from plants

Volatile oils from leaves of OS and OK were extracted by steam distillation [23]. This procedure gave an average yield of 0.2 mls of volatile oils per kilogram of fresh leaves of *Ocimum *plants. The extracted oil was stored in airtight bottle and kept at 4°C for later experiments.

### Formulation of repellents

Four repellent formulations were prepared as follows: (1) 20% DEET (N, N,-diethyl-3-methylbenzamide) in glycerine with acetone as solvent; (2) 20% OS extract in glycerine with acetone as solvent; (3) 20% OK extracts in glycerine with acetone as solvent and (4) a mixture of glycerine with acetone was used as control. The products were applied on human skin as commonly done for normal body oil.

### Impregnation of materials with extracts

The netting materials used for cone bioassay and tunnels tests [24] were treated at different concentrations: 75 mg/m^2, ^100 mg/m^2^, 200 mg/m^2^, 250 mg/m^2 ^and 500 mg/m^2^. These dosages were calculated using the proportion of percentage of the targeted constituent of the crude essential oil composition. The dosage is comparable to concentration of pyrethroid used for net treatment approved for community use [25]. The filter papers used for susceptibility test were treated with *OS, OK *and citronella oil at concentrations of 75 mg/m^2, ^100 mg/m^2^, 200 mg/m^2^, 250 mg/m^2 ^and 500 mg/m^2^. The treated materials were stored at room temperature until the time of experiment.

### Tunnel chamber experiment

Tunnel chamber (90 cm × 30 cm × 30 cm), made up of wood frames and 4 mm glasses was used to evaluate repellence, mortality and feeding inhibition of plant extracts against three mosquito species. It had three sections of equal dimensions (30 cm × 30 cm × 30 cm). The first partition, a release chamber, is where host-seeking mosquitoes are released at the beginning of experiment. The middle chamber is separated from the bait chamber by netting material with nine holes of 1 cm diameter which allows mosquito to get into a bait chamber. The third is a bait chamber, where bait (e.g. a rabbit) is fixed in a wooden cage. This tunnel chamber, modified from Chandre and others [26], is designed to imitate the setting of indigenous rural African houses, where in the majority of households, animals like chickens, cattle and goats are kept indoors. The test procedure and analysis methods used are described elsewhere [27].

### Laboratory studies

#### Cage tests

The hands of two volunteers were exposed to mosquitoes in cages (30 cm × 30 cm × 30 cm) for one hour. Test hand was treated and control hand was untreated. The same volunteer was used to evaluate all three formulations (DEET 20%; OS 20% and OK 20%) in order to avoid bias related to preferential attractiveness to mosquitoes. Volunteers were exposed to different treatments with different mosquito species. Each exposure had five replicates of 25 mosquitoes, making a total of 125 mosquitoes used per treatment per species. All tests were conducted at a room temperature of 27 ± 2 degree centigrade and relative humidity of 78%.

#### Susceptibility bioassays

The bioassay tests were done on three mosquito species (*An. gambiae s.s, An. arabiensis*, and *Cx. quinquefasciatus*) using susceptibility kit and standard methods of World Health Organization [28]. Mosquito were exposed to filter papers treated with different dosages (75 mg/m^2, ^100 mg/m^2^, 200 mg/m^2^, 250 mg/m^2 ^and 500 mg/m^2^) of citronella and extracts of essential oils from OK and OS plants. Untreated filter papers were used as control.

Four replicates and one control for each dosage were done for each mosquito species tested. In total 125 (100 in four replicates of treatment and 25 in control) unfed female mosquitoes were tested for each species. Mosquitoes were exposed for one hour then transferred to holding chamber where mortality and recovery (i.e. immediate mortality and 24 hours mortality) were scored. Also time taken to knockdown 90% of the population (KD_90_) and 95% confidence interval were calculated per treatment.

#### Contact or cone bioassays

Netting material treated with six different dosages (75 mg/m^2, ^100 mg/m^2^, 200 mg/m^2^, 250 mg/m^2 ^and 500 mg/m^2^) of Ocimum extracts and citronella were used for the cone bioassay. Five unfed female mosquitoes were exposed to treated material for three minutes, ten replicates per each test. The cones were held on treated surface and the control on untreated surface. The exposed mosquitoes were taken for observations to record immediate mortality, 24 hours mortality and recovery after 24 hours.

#### Ethical considerations

Ethical clearance was reviewed and granted by Ethics committee of Tumaini University, Moshi Tanzania. The purpose of study was elaborated to the head of households before the survey and participation was voluntary.

### Statistical analysis

The percentages of blood feeding inhibition of mosquitoes for cage test, knockdown effect and mortality for both contact and WHO susceptibility tests, repellence and feeding inhibition for Tunnel test were computed using excel spreadsheet. The percentage protection (feeding inhibition, mortality or knockdown effect) was estimated by Abbot formula as PE = (N_C _- N_T_)/N_T _× 100%, where N_C _and N_T _are the number of mosquito on control and on treatment, respectively [18,19]. Abbot formula was used to correct for mosquitoes feeding responses by different treatments in Tunnel and Cage experiments.

In contact and susceptibility tests, percentage mortality and recovery rates for Ocimum extracts (OS and OK) against citronella were calculated. Knockdown effect was determined as the proportion of mosquito knockdown at a particular observation. Data were subjected to one way analysis of variance (ANOVA) and mean percentage knockdown between treatments and against DEET for each of three mosquito species were compared by sample T – tests and significance level was determined at P < 0.05.

## Results

A total number of 120 head of households were interviewed at lower Moshi covering Rau Kati (30 households), Mtakuja (31), Chekereni (30) and Mabogini (29) villages to assess the social demographic data (Table 1). In these villages, plant species commonly used as mosquito repellents were *Ocimum species *(56.6%), *Azadirachta indica *(30.1%), *Eucalyptus globules *(11.6%) and *Lantana camara *(1.7%).

**Table 1:** Summary of socio-demographic data from four villages at Lower Moshi, northern Tanzania, based on 120 households

**Evaluated factor**	**Assessed variable**	**Number of respondent (%)**
Sex of head of household interviewed	Males	83(61%)
	Females	37(39%)
Number occupants	Mean per house	5
Level of education	Below primary school	26(22%)
	With primary School	70(58%)
	Above primary School	24(20%)
House type	Mud wall and thatch roof	75(63%)
	Brick walls and iron roof	45(37%)
Occupation	Peasant (Small farmers)	87(73%)
	Employed	20(17%)
	Business	13(11%)

Methods of application were mostly incense burning/smouldering considered to offer effective protection and application was mostly done around 7 pm to 10 pm in 60% of households interviewed of which majority were small scale farmers (73%). The plant parts mostly used were leaves (70%), barks (10%), mixed plant parts (13%) or roots (7%).

### Knockdown effect based on exposure to filter papers impregnated with plant extract

The knockdown effect induced by the two Ocimum plants and citronella ranged from 35% to 50% in all species of mosquitoes tested. This effect however, varied significantly (*P *< 0.05 – P > 0.05) among the three mosquito species tested. Citronella had the highest knockdown effect followed by OS and OK. At a dose of 30 mg/m^2^, citronella achieved over 50% knockdown within 10 minutes, while extracts of test plants (OS and OK) achieved between 35% to 45% knockdown for all species tested. There was no significant difference between the knockdown effect due to citronella and OS in all species tested. The OK showed the lowest knockdown effect in all species, whereas citronella scored a significantly high knockdown effect (P = 0.001). The KD_90 _results for all species against plant extracts are summarized in Table 2.

**Table 2:** The KD_90 _of different plant extracts based on three mosquito species

Treatment/Species	*An. gambiae ss* (KD_90 _in Min.)	*An. arabiensis * (KD_90 _in Min.)	*Cx. Quinquefasciatus* (KD_90 _in Min.)
OK	19.1	19.5	15.2
OS	14.9	20.7	20.9
Citronella	12.9	10.3	16.9

In general, the knockdown efficacy of OS was significantly higher on *An. gambiae *(P = 0.027), whereas that of OK was significant on *An. arabiensis *(P = 0.007). On the other hand, the knockdown effect of both OS (P = 0.223) and OK (0.045) was not significant on *Cx. quinquefasciatus*.

### Mosquito mortality in experiments with treated netting materials (contact bioassays)

In all tests, citronella induced the highest mortality rate on both species, followed by OS on Anopheles species and OK on Culex species. Mortality rate was high in *An. gambiae *s.s up to cut off point (30 mg) for all the test products. Citronella induced the highest mortality (56.6%) followed by OS (47.5 %) and the lowest was OK (43.3%).

At a concentration of 30 mg, mortality caused by exposure to citronella were high in *An. arabiensis *(67.7%, P < 0.001) followed by *An. gambiae ss *(56.7%, P < 0.001) and *Cx. quinquefaciatus *63.7% (P = 0.001).

In *An. arabiensis*, mortality induced by citronella was 67.7% (P < 0.0001); 62.0% for OS (P = 0.006) and 55% (P = 0.051) for OK. Mortality induced by both citronella and OS are comparable and significantly higher than mortality caused by OK.

In *An. gambiae *s.s., citronella achieved the highest mortality of 56.7% (P < 0.001) followed by OS 47.4% (P = 0.001) and OK 43.3% (P = 0.054) at a dosage of 30 mg. The citronella caused more than 50% mortality, but the effect of both OS and OK was comparable and below 50%.

In *Cx. quinquefasciatus*, citronella caused the highest mortality 63.7% (P = 0.001) followed by 56.3% for OK (0.012) and 46.3% for OS (P = 0.062). Mortality due to OK and citronella was comparable and significantly higher on Culex mosquitoes. With increased dosage to 50 mg, there was no significant increase in mortality in all species and in all treatments.

### Biting inhibition of mosquitoes

**Figure 1:**
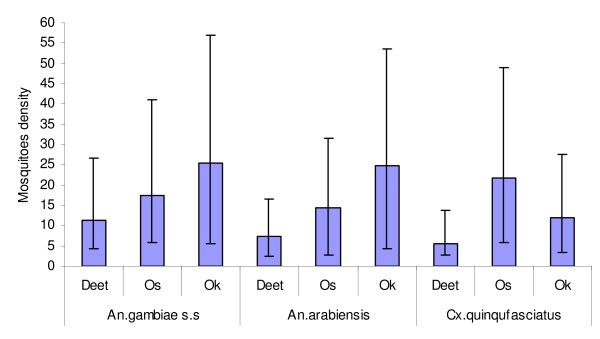
Biting success exhibited by three mosquito species treatments into twenty percent concentration of DEET, OK and OS.

This present finding of cage experiments which compared the number of mosquitoes landed on treated and untreated arms of volunteers. The highest biting inhibition rate against all tested mosquito species was achieved by DEET (ranging from 88.7% to 92.5 %) followed by OS (83.5% to 88.9%) and OK (71.2% to 85.3%). Among the natural products tested, variation in biting inhibition within the species was observed; OS was more inhibiting for the *An. gambiae s.s *and *An. arabiensis *whereas the OK was efficient in inhibiting *Cx. quinquefasciatus*. The feeding inhibition caused by OS (P < 0.001) was significant higher than OK (P = 0.045) on both species of Anopheles tested. Likewise OK induced significant feeding inhibition on *Cx. quinquefaciatus*, but overall DEET gave the highest biting inhibition (Figure 1).

### Tunnel experiments

**Figure 2:**
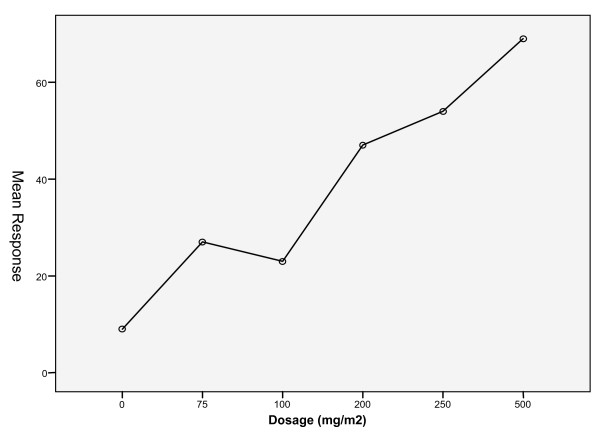
The mean response of mosquito repelled/inhibited from feeding in the tunnel experiment against different dosages (0 mg/m^2 ^(Control), 75 mg/m^2^, 100 mg/m^2^, 200 mg/m^2^, 250 mg/m^2^, 500 mg/m^2^) of plant extracts.

All measured protective effects namely repellence, feeding inhibition and mortality rates of test extracts (OS or OK) were compared against CT, a standard repellent to obtain significance levels. The trend in repellency effect, feeding inhibition and mortality were found to increase with dosage (ranged from 75 mg to 500 mg/M^2^) of extracts (Figure 2) impregnated on netting materials. The dosage of 500 mg/m^2 ^was found to be the most effective and this was used to compare the effects of different exposures.

### Citronella

In the tunnel experiment, the overall repellence effect induced by netting material treated with citronella against mosquitoes entering the bait chamber was 81% for *An. gambiae *s.s, 85% for *An. arabiensis *and 98 % for *Cx. quinquefasciatus*. There was feeding inhibition of 93% for *An. gambiae *s.s, 96 % for *An. arabiensis *and 100% for *Cx. quinquefasciatus*. Mortality rates were 63% for *An*. *gambiae *s.s, 69% for *An*. *arabiensis *and 65% for *Cx. quinquefasciatus*. Notably, all test effects, repellence (Table 3), feeding inhibition (Table 4) and morality rates (Table 5) induced by citronella were significant for all three species of mosquitoes.

**Table 3:** Repellency effects against *An. gambiae, An. arabiensis *and *Cx. quinquefasciatus *induced by 500 mg/m^2 ^of each of citronella and two extracts of *Ocimum suave *and *Ocimum kilimandscharicum*.

	*An. gambiae *(N = 100)		*An. arabiensis *(N = 100)		*Cx. quinquefaciatus*(N = 100)	
	% Repellency	P-value*	% Repellency	P-value*	% Repellency	P-value*

Citronella	81	0.000	85	0.000	98	0.000
OS	81	0.020	89	0.010	91	0.07
OK	73	0.050	75	0.070	82	0.040

*The paired samples T-test was used to compare the means

**Table 4:** Feeding inhibition of *An. gambiae, An. arabiensis *and *Cx. quinquefasciatus *induced by 500 mg/m^2 ^of each of standard repellent citronella and two extracts of *Ocimum suave *and *Ocimum kilimandscharicum*.

Test Product	*An. gambiae * (N = 100)		*An. Arabiensis* (N = 100)		*Cx. quinquefasciatus* (N = 100)	
	
	% Feeding inhibition	P- value*	%Feeding inhibition	P- value*	% Feeding inhibition	P- value*
Citronella	93	0.000	96	0.000	100	0.000
OS	88	0.012	90	0.012	100	0.000
OK	77	0.060	90	0.012	99	0.003

*The paired samples T-test was used to calculate the p-values

**Table 5:** Mortality rates scored for *An. gambiae*, *An. arabiensis *and *Cx. quinquefasciatus *induced by 500 mg/m^2 ^for each of standard repellent Citronella and two extracts of *Ocimum suave *(OS) and *Ocimum kilimandscharicum *(OK).

Test	*An. gambiae *(N = 100)		*An. arabiensis *(N = 100)		*Cx. quinquefasciatus *(N = 100)	
Product	% Mortality	P-value*	% Mortality	P-value*	% Mortality	P-value*

Citronella	63	0.000	69	0.000	65	0.000
OS	50	0.009	58	0.001	67	0.039
OK	47	0.001	52	0.040	65	0.001

*The paired samples T-test was used to calculate the p-values.

#### Ocimum suave

Overall repellence induced by OS was 81% for *An. gambiae *ss, 89% for *An. arabiensis *and 91% for Cx.*quinquefasciatus*. High level of feeding inhibition was observed, indeed 88% of *An. gambiae *ss, 90% of *An. arabiensis *and 100% of *Cx. quinquefasciatus *could not feed on the bait. Mortality rates due to OS were 50% for *An. gambiae *s.s, 58% for *An. arabiensis *and 67% for *Cx. quinquefasciatus*. Notably, repellence effect (Table 3), feeding inhibition (Table 4) and mortality rates (Table 5) induced by OS were significant for all three species of mosquitoes tested.

#### Ocimum kilimandscharicum

An overall repellence effect induced by OK was 73%, 75% and 82% for *An. gambiae *s.s, *An. arabiensis *and *Cx. quinquefasciatus *respectively. Likewise feeding inhibition was 77% for *An. gambiae *s.s, 75 % for *An. arabiensis *and 99% for *Cx. quinquefasciatus*. Mortality scored were 47%, 52% and 56% for *An. gambiae *s.s, *An. arabiensis *and *Cx. quinquefasciatus *respectively. Repellence effect (Table 3) and feeding inhibition (Table 4) induced by OK was significant for *An. arabiensis *and *Cx quinquefasciatus *only. However, mortality rates were significant higher for all three species of mosquitoes tested (Table 5).

## Discussion

In north-eastern Tanzania, the use of plants repellents to reduce human vector contact is a common practice among village communities. The major plants used as repellents are Ocimum species (*Ocimum kilimandscharicum *and *Ocimum suave*), *Eucalyptus species*, *Lantana camara *and *Azadirachta indica*. The majority of interviewed villagers admitted that they could not afford to use synthetic commercial mosquito repellents or insecticides because of high cost and or discouraged by poor performance of some of the products. Similar finding have been reported elsewhere in African, such as Guinea Bissau and Kenya, where the majority of the villagers could not afford synthetic commercial mosquito insecticides due to poverty [18,29].

Majority of the interviewed households (66.7%) showed preference to Ocimum species than other anti-mosquitoes plants. Ocimum plants are common as the post-harvest weed in the area around these villages. Most common method of application is burning of plant leaves for protection before going to bed, hanging the repellent plants inside the house concurring with other reports from Africa [16,30]. Application of repellents was mostly done between 7 pm to 10 pm, such timing corresponds with the mosquito active biting cycle in the evening. Curtis and others [31] recorded up to a ten fold reduction in mosquito biting close to smouldering *Hyptis suaveolens; *but there was not any reduction of mosquito biting in a hut with fresh *H. suaveolens*. Extracts of Ocimum plants have shown significant protection against the malaria vectors in different areas of Africa [16,19].

### Knockdown effect on tested mosquitoes

At a standard dosage of 30 mg/m^2^, the knockdown effect induced by OS, OK and citronella ranged from 35% to 50%, this effect however, varied significantly (*P *< 0.05 – P > 0.05) among the three species tested. Similar results were reported on a test done with *Callosobruchus maculates *of which the knockdown effect varied with concentrations of extracts [32].

The knock down effect (KD_90_) was calculated to estimate the population of mosquitoes that will be knocked down at the standard time of ten minutes as recommended for insecticides used for the space spraying such as aerosols [33]. The KD_90 _accounts for the efficacy of insecticide to knockdown 90% of the population in ten minutes. Such insecticides therefore need to have the ability to knockdown 90% of the insect population in ten minutes so as to be considered for space spraying as recommended by WHO [33]. In this study, Citronella fulfilled this criterion; the OS was second best with KD_90 _of 43 min on *An. arabiensis *and 42 min on *Cx. quinquefasciatus *knockdown within and 42 minutes respectively.

Variation in knockdown rates among OS and OK between various species of mosquito is probably due to the effect of the major active ingredients found in the extracts [29]. The knockdown effect of OK might has been attributed by the presence of camphor found in high concentration in its essential oil. The camphor has a terpenoids in their hydrocarbon structure, which confers a common property of hydrophobicity [34]. Many hydrophobic compounds are associated with the protein deactivation leading to enzyme inhibition [35]. The knockdown effect of OS might have been attributed with the high amount of eugenol (60%) found in its essential oils. Likewise, OS extract contains linalool (1.4%), a compound known to act as a reversible inhibitor of acetyl cholinesterase, capable of disrupting the function of neurotransmitter in insects like mosquito, hence inducing knockdown effect [35].

#### Mortality rates on tested mosquito

Plant secondary metabolites are thought to act as toxicants or deterrents [17]. This study revealed that, essential oil extracts tested had good insecticidal activity against mosquito and the effect is dosage dependant [25].

Citronella, OS and OK impregnated in filter papers and netting materials were toxic to all three species of mosquitoes tested. There was 100 percent knockdown due to exposure to Ocimum extracts. Mortality of both Anopheline and Culicine was found to increase proportionately with increase in concentration of the plant products; either in filter papers or netting material. Similar effect of dosage dependant mortality has been found in permethrin-treated materials [25].

The toxicity and bioactivity of OS may be attributed with high concentration of eugenol, a compound with phenolic structure [36] and the presence of other compounds such as linalool. Phenols are generally known to be important sources of potent insecticides, fungicides, bactericides and herbicides for pest control. These results suggest a potential of OS to be used as repellents and toxicant agent against mosquitoes. Similarly, the repellent effect of eugenol against insects has been demonstrated elsewhere Hassanali *et al *[34].

Repellence activities of Ocimum plants against insects have been reported elsewhere. In Rwanda, farmers protect farm-stored edible beans (*Phaseolus vulgaris*) against insect damage by using leaves of *Ocimum canum*. Linalool is the major component of the essential oil of this annual mint, representing 60–90% of the total volatile collected [37] and act as a reversible competitive inhibitor of acetyl cholinesterase [35].

Extracts from OK, with high concentration of camphor, induced low mortality in tested mosquitoes in comparison with citronella and OS. According to Ryan and Byrne [35], the effect of camphors is attributed to toxicity of several terpenoids representing a range of functional groups including pulegone (ketone), linalool (alcohol) and 1,8-cineole (ether) against pests to their reversible competitive inhibition of acetylinecholinesterase by apparently occupying the hydrophobic sites of the enzymes active centre. Although, OK has high concentration of camphor, its protection effects against insect biting may be attributed to other minor toxic components such as 1,8-cineole (7%), limonene (6%) and linalool (0.5%). Since concentration of linalool in OS (1.4%) is much higher than in OK (0.5%), a significant mortality induced by OS could be attributed to the high amount of linalool.

### Tunnel experiments

In the tunnel experiments, the feeding inhibition, repellence effect and mortality response patterns measured on *An. gambiae, An. arabiensis *and *Cx quinquefasciatus *was the most effective at the dosage of 500 mg/m^2^. Overall Ocimum extracts in treated material at different dosage provided substantial repellence effects on tested mosquitoes. The performance of extracts from OS, with mortality range from 50% to 67%, was the second best to the commercial product (citronella) and these results are comparable to report elsewhere [29]. Permethrin, a commonly used pyrethroid insecticides evaluated at similar dosages of 500 mg/m^2 ^in tunnel experiments against *An. gambiae *s.s induced mortality rate of around 80%. At a lower dosage of 100 mg/m^2^, mortality was around 58% [25]. The performance efficacy of crude extracts of OS and OK, measured in terms of repellence, mortality and feeding inhibition effects, at the dosage of 500 mg/m^2 ^have shown possibility of increasing protection against malaria vectors [38]. Since Ocimum plants are abundant and locally available, the community use of such repellent plants to compliment existing control measures is feasible, such as treating mosquito nets once a month in rural areas where affordability of the ITNs is restricted. Meanwhile, more research on formulation of Ocimum extracts suitable for community use is required.

Notably, the effect of OS and OK extracts varied by dosage and the mosquito species. The differences in the active phytochemicals such as eugenol, camphor and other compounds influence the protective effect of Ocimum plants. In particular variation in the concentration of linalool component found in OS (1.4%) and OK (0.4%) could explain difference in mortality rates induced by the two plants [39].

## Conclusion

The protective effects in terms of feeding inhibition, knockdown effect and mortality by *O. suave *and *O. kilimandscharicum *plant extracts is significant particularly against mosquito biting. The community-wide use of such repellent plants has potential to compliment existing control measures, such as treating mosquito nets once a month in areas where affordability of the ITNs is restricted. However, the duration of effect of Ocimum essential oils is compromised due to its high volatility and hence further investigation should focus on developing formulation applicable for community application. Specifically, such formulation can be promoted for protection against early biting cycle of mosquito in the evening before going to bed and for those exposed to early morning biting cycle. Likewise assays to quantify and determine contribution of different active ingredients of *O. suave *and *O. kilimandscharicum *extracts to its protective effect should be investigated because this may lead to discovery of novel compound(s) with desired insecticidal activities.

## Competing interests

The authors declare that they have no competing interests.

## Authors' contributions

EJK and FWM conceived and designed the study. EJK and EAT participated in the analysis and interpretation of data and contributed to the drafting of the manuscript. AL prepared the questioner for the sociological survey. EAT, FT and AMM carried out data analysis and interpretation, and were involved in the drafting of the manuscript. EJK, JK, JM, JM, CM, FT, EL, MM, RM and GC reared mosquitoes and performed experiments, participated in the analysis and interpretation of results, and was involved it the drafting of the manuscript. All authors read and approved the final copy of manuscript.

## References

[B1] Nathan SS, Kandaswamy K, Kadarkarai M (2005). Effect of neem limonoids on the malaria vector *Anopheles stephensi *Liston (Diptera: Culicidae). Acta Trop.

[B2] Snow RW, Guerra CA, Noor AM, Myint HY, Hay SI (2005). The global distribution of clinical episodes of *Plasmodium falciparum *Malaria. Nature.

[B3] Brooke BD, Humb RH, Koekemorer LL, Dossou-Yovo J, Coetzee M (2000). Evaluation of a polymerized chain reaction assay for detection of pyrethroid insecticide resistance in the malaria vector species of the *An. gambiae *complex. J Am Mosq Control Assoc.

[B4] Lengeler C, Smith TA, Armstrong-Schellemberg J (1997). Focus on the effect of bednets on malaria control morbidity and mortality. Parasitol Today.

[B5] Beier JC, Killeen GF, Githure JI (1999). Short report: entomologic inoculation rates and *Plasmodium falciparum *malaria prevalence in Africa. Am J Trop Med Hyg.

[B6] Casmiro S, Coleman M, Mohloai P, Hemingway J, Sharp B (2006). Insecticide resistance in *Anopheles funestus *(Diptera: Culicidae) from Mozambique. J Med Entomol.

[B7] Binka FN, Adongo P (1997). Acceptability and use of insecticide impregnated bed nets in Northern Ghana. Trop Med Int Health.

[B8] Snow RW, McCabe E, Mbogo CNM, Molyneux CS, Some ES, Mung'ala VO, Nevill CG (1999). The effect of delivery mechanism on the effect of bed net re-impregnation in Kilifi district, Kenya. Health Policy Plan.

[B9] Allaii JA, Hawley WA, Kolczak MS, ter Kuile FO, Gimnig JE, Vulule JM, Odhacha A, Oloo AJ, Nahlen BL, Phillips-Howard PA (2003). Factors affecting use of permethrin-treated bed nets during a randomized controlled trial in western Kenya. Am J Trop Med Hyg.

[B10] Allaii JA, Borne HW Van Den, Kachur SP, Shelley K, Mwenesi H, Vulule JM, Hawley WA, Nahlen BL, Phillips-Howard PA (2003). Community reactions to the introduction of permethrin-treated bed nets for malaria control during a randomized controlled trial in western Kenya. Am J Trop Med Hyg.

[B11] Das NG, Baruah I, Talukdar PK, Das SC (2003). Evaluation of botanicals as repellents against mosquitoes. J Vector Borne Dis.

[B12] Chogo JBA, Crank G (1981). Chemical composition and biological activity of the Tanzania plant *Ocimum suave*. J Nat Prod.

[B13] Ansari MA, Vasudevan P, Tandon M, Razdan RK (2000). Larvicidal and mosquito repellent action of peppermint *(Mentha piperita*) oil. Biores Technol.

[B14] Kilonzo BS, Ngomuo AJ, Sabuni CA, Mgoe GF (2001). Effect of *Azadirachta indica *(NEEM) extract on livestock fleas in Morogoro district, Tanzania. Insect Science Applic.

[B15] Okumu FO, Knols BG, Fillinger U (2007). Larvicidal effects of a neem (*Azadirachta indica*) oil formulation on the malaria vector *Anopheles gambiae*. Malar J.

[B16] Seyoum A, Killeen GF, Kabiru EW, Knols BG, Hassanali A (2003). Field efficacy of thermally expelled or live potted repellent plants against African malaria vectors in western Kenya. Trop Med Int Health.

[B17] Tawatsin A, Wratten SD, Scott R, Thavara U, Techadamrongsin Y (2001). Repellency of volatile oil from plants against three mosquito vectors. J Vector Ecol.

[B18] Palsson K, Jaenson TG (1999). Comparison of plant products and pyrethroid-treated bed nets for protection against mosquitoes (Diptera: Culicidae) in Guinea Bissau, West Africa. J Med Entomol.

[B19] Waka M, Hopkins RJ, Glinwood R, Curtis CF (2006). The effect of repellents *Ocimum forskolei *and DEET on the response of *Anopheles stephensi *to host odours. J Med Vet Entomol.

[B20] Hebbalkar DS, Hebbalkar GD, Sharma RN, Joshi VS, Bhat VS (1992). Mosquito repellent activity of oils from *Vitex negundo *Linn. Leaves. Indian J Med Res.

[B21] Dua VK, Gupta NC, Pandey AC, Sharma VP (1996). Repellency of *Lantana camara *(Verbenaceae) flowers against *Aedes *Mosquitoes. J Am Mosq Control Assoc.

[B22] National census report 2002. http://www.tanzania.go.tz/censusdb/index.html.

[B23] Peter JH, Amala R (1998). Laboratory handbook for the extraction of natural extracts.

[B24] World Health Organization Guideline for laboratory and field testing of mosquito larvicides WHO/CDS/WHOPES/GCDPP/200513, Geneva, Switzerland.

[B25] Corbel V, Chandre F, Brengues C, Akogbeto M, Lardeux F, Hougard JM, Guillet P (2004). Dosage -dependant effect of permethrin-treated nets on the behaviour of the *Anopheles gambiae *and selection of pyrethroid resistance. Malar J.

[B26] Chandre F, Damet F, Duchon S, Finot L, Manguin S, Carnevale P, Guillet P (2000). Modifications of pyrethroid effects associated with kdr mutation in *Anopheles gambiae*. Med Vet Entomol.

[B27] Pitawasat B, Choochote W, Tuetun B, Tippawangkosol P, Kanjanapothi D, Jitpakdi A, Riyong D (2003). Repellency of aromatic turmeric *Curcuma aromatica *under laboratory and field conditions. J Vector Ecol.

[B28] World Health Organization (1996). Report of the WHO informal consultation on the evaluation and testing of the insecticides CTD/WHOPES/IC/961, Control of Tropical Diseases Division.

[B29] Odalo JO, Omolo MO, Malebo H, Angira J, Njeru PM, Ndiege IO, Hassanali A (2005). Repellency of essential oils of some plants from the Kenyan coast against *Anopheles gambiae*. Acta Trop.

[B30] Bockarie MJ, Service MW, Barnish G, Momoh W, Salia F (1994). The effect of woodsmoke on the feeding and resting behaviour of *Anopheles gambiae s.s*. Acta Trop.

[B31] Curtis CF, Lines JD, Baolin L, Renz A, Curtis CF (1991). Natural and synthetic repellents. Control of disease vectors in the Community.

[B32] Keta SM, Vincent C, Schmidt JP, Ramaswamy S, Belanger A (2000). Effect of various essential oils on *Callosobruchus maculates *(F.) (Coleoptera: Bruchidae). J Stored Product Research.

[B33] World Health Organization (1975). Manual on practical entomology in malaria. Part II. WHO Division of Malaria and Other Parasitic Diseases, Geneva.

[B34] Hassanali A, Lwande W, Ole-sitayo N, Moreka L, Nokoe S, Chapya A (1990). Weevil repellent constituents of *Ocimum suave *leaves and *Eugenia caryophyllata *cloves used as grain protectant in parts. E Afr Disc Innov.

[B35] Ryan MF, Bryan O (1998). Plant-insect co-evolution and inhibition of acetyl cholinesterase. J Chem Ecol.

[B36] Cremlyn R (1987). Pesticides: preparation and mode of action.

[B37] Weaver DK, Dunkel FV, Ntezurubanza L, Jackson LL, Stock DT (1991). The efficiency of Linalool, a major component of freshly milled *Ocimum canum *(Sims) (Lamiaceae), for protection against stored product Coleoptera. J Stored Prod Res.

[B38] Hill N, Lenglet A, Arnéz AM, Carneiro I (2007). Plant based insect repellent and insecticide treated bed nets to protect against malaria in areas of early evening biting vectors: double blind randomised placebo controlled clinical trial in the Bolivian Amazon. BMJ.

[B39] Jembere B, Obeng-Ofori D, Hassanali A, Nyamasyo GN (1995). Products derived from the leaves of *Ocimum kilimandscharicum *(Labiatae) as post-harvest grain protectants against the infestation of three major stored product insect pests. Bull Entomol Res.

